# The Association of Cysteine with Obesity, Inflammatory Cytokines and Insulin Resistance in Hispanic Children and Adolescents

**DOI:** 10.1371/journal.pone.0044166

**Published:** 2012-09-11

**Authors:** Amany K. Elshorbagy, Maria Valdivia-Garcia, Helga Refsum, Nancy Butte

**Affiliations:** 1 Department of Pharmacology, University of Oxford, Oxford, United Kingdom; 2 Department of Physiology, Faculty of Medicine, University of Alexandria, Alexandria, Egypt; 3 Institute of Basic Medical Sciences, Department of Nutrition, University of Oslo, Oslo, Norway; 4 USDA/ARS Children’s Nutrition Research Center, Department of Pediatrics, Baylor College of Medicine, Houston, Texas, United States of America; University of Milan, Italy

## Abstract

**Context:**

Plasma total cysteine (tCys) independently relates to fat mass in adults. Dietary cyst(e)ine promotes adiposity and decreases glucose tolerance in some rodent models, but alleviates insulin resistance in others.

**Objective:**

To investigate whether the association of tCys with body fat extends to children at particular risk of obesity, and whether tCys is associated with insulin resistance and obesity-associated inflammation.

**Methods:**

We explored the cross-sectional relations of fasting plasma tCys and related metabolites with body composition measured by dual-energy X-ray absorptiometry in 984 Hispanic children and adolescents aged 4–19 years from the Viva La Familia Study. Linear and logistic regression and dose-response curves were used to evaluate relations of tCys with obesity, insulin resistance and inflammatory markers including interleukin-6 (IL-6), tumor necrosis factor-alpha (TNF-α), monocyte chemoattractant protein-1 (MCP-1) and C-reactive protein (CRP).

**Results:**

tCys, methionine and total homocysteine (tHcy) increased with age. Upper tCys quartile was independently associated with a 5-fold increased risk of obesity (95% CI 3.5–8.0, *P*<0.001), and 2-fold risk of insulin resistance (95% CI: 1.6-5.0, *P*<0.001; adjusted for body fat%). Within the overweight/obese subgroup, but not in normal-weight children, tCys accounted for 9% of the variability in body fat% (partial r = 0.30, *P*<0.001; adjusted for age and gender). tCys correlated positively with serum non-esterified fatty acids and leptin, partly independent of body fat, but was not associated with serum IL-6, TNF-α or MCP-1. A positive correlation with CRP disappeared after adjustment for BMI.

**Conclusion:**

tCys is independently associated with obesity and insulin resistance in Hispanic children and adolescents, highlighting a previously underappreciated link between the sulfur amino acid metabolic pathway and obesity and cardiometabolic risk.

## Introduction

Emerging evidence from knockout studies points to the involvement of the sulfur amino acid (SAA) metabolic pathway in regulation of body weight and glucose homeostasis. Homozygous deletion of cystathionine beta synthase (CBS) enzyme, which catalyses the first step of cysteine synthesis from homocysteine, reduces body fat in mice (Figure-S1) [1]. Knockout mice lacking betaine-homocysteine methyltransferase (BHMT), one of the enzymes that remethylate homocysteine to methionine, have increased energy expenditure and insulin sensitivity, and are resistant to diet-induced obesity [2]. A similar phenotype is observed in mice with a defect in glutathione synthesis due to deficiency of the glutamate-cysteine ligase modifier subunit [3], [4], and in wild-type rats fed a methionine-restricted diet [5]. Common to all these models is decreased cysteine synthesis and/or plasma cysteine [1], [3], [5], [6]. Profound hepatic suppression of stearoyl coenzyme A desaturase-1 (SCD1), a key lipid synthesizing enzyme and checkpoint in development of obesity [7], is also seen in all models except for BHMT−/− where it has not been tested.

L-cysteine supplementation reverses the methionine restriction-induced suppression of SCD1, and restores fat gain in rats [8]. In mice, high cystine intake lowers energy expenditure and decreases glucose tolerance, with up-regulation of lipogenic and diabetogenic enzymes [9]. Collectively, these data, supported by *in vitro* and *in vivo* studies reviewed in [10], and human data listed below, raise the hypothesis that cysteine or a closely related factor may be obesogenic. Not all evidence fits with this hypothesis though. In CBS−/− mice, supplementation of the cysteine donor N-acetylcysteine, fails to restore body fat [1]. Also knockout mice lacking cysteine dioxygenase, the enzyme that initiates cysteine catabolism, have high plasma cysteine but low body mass [11].

In humans, increased plasma total cysteine (tCys) is associated with higher fat mass (FM) and obesity [12], [13]. Increase of tCys over 6 years was independently associated with 2-kg higher FM at follow-up [12], and 6-year-change in tCys correlated with change in BMI [14]. These associations were unique to cysteine and not shared by the cysteine precursors methionine and homocysteine, or the cysteine products taurine and glutathione [15]. Studies of these associations have thus far been conducted in adults of European descent. While a causal link between cysteine and human obesity is yet to be demonstrated, it would be interesting to know whether the association of cysteine with fat mass is relevant also to younger subjects and other ethnic groups at higher risk of obesity and obesity-related disorders.

Obesity triggers a host of cardiometabolic complications, the hallmark of which is insulin resistance, while only a third of obese individuals remain “metabolically healthy” [16]. Insulin resistance in obesity is thought to be mediated via non-esterified fatty acids (NEFA) and proinflammatory factors including tumor-necrosis-factor-alpha (TNF-α), interleukin-6 (IL-6) and monocyte chemoattractant protein-1 (MCP-1) [17]. A potential role of amino acid metabolism in pathogenesis of insulin resistance and diabetes was suggested by findings that elevation of several amino acids was associated with 5-fold risk of developing diabetes 12 years later [18]. Plasma tCys was not measured, but elevated plasma cystine (the non-protein bound dimer constituting about 25% of plasma tCys), was observed in cross-sectional metabolomic studies of insulin resistance and diabetes [19], [20]. Due to its association with body fat, tCys might be a good predictor of insulin resistance. However, cysteine has insulin-like effects *in vitro* [21], and alleviates sucrose-induced insulin resistance [22], suggesting that elevated tCys in humans might be associated with metabolically healthy obesity.

In the present study we explored a) the associations of fasting plasma tCys and related SAA with body fat% in the Viva La Familia cohort of Hispanic children and adolescents [23]; and b) the association of tCys with circulating NEFA, inflammatory cytokines and insulin resistance.

## Methods

### Subjects

1030 children from 319 families were enrolled in the Viva La Familia Study in Houston, TX, between 2000 and 2004 [23]. Each family was selected based on an obese proband aged 4-19 years using a bivariate ascertainment scheme, i.e., ≥95^th^ percentile for BMI and ≥85^th^ percentile for FM [23]. Families were required to have ≥3 children aged 4–19 years. The current study is confined to 984 children for whom plasma samples were available for biochemical analysis of SAA. Enrolled children and parents gave written informed consent or assent. The protocol, which covered future use of stored blood samples, was approved by the Institutional Review Boards for Human Subject Research at Baylor College of Medicine and Affiliated Hospitals and Southwest Foundation for Biomedical Research.

### Study Variables

#### Anthropometrics, body-composition and tanner staging

Weight was measured with a digital balance to the nearest 0.1 kg, and height was measured to the nearest 1 mm with a stadiometer. Body composition was determined by dual-energy X-ray absorptiometry (DXA) with a Delphi-A whole-body scanner (Hologic Inc, Waltham, MA), using the manufacturer’s software (version 11.2). Total body and regional estimates of FM and fat-free mass (FFM) were obtained by using the manufacturer’s software (version 11.2).

Waist-height ratio [24] and the ratio of trunk fat to total fat were used as measures of central adiposity. Overweight and obese were defined according to recent recommendations (2010) of the Centre for Disease Control and Prevention (CDC) as BMI percentile≥85^th^ and ≥95^th^ percentile for age [25].

Tanner stages of sexual maturation based on pubic hair and breast and male genital development, which had been illustrated with drawings, were self-reported.

#### Diet and physical activit*y*

Multiple-pass 24-hour dietary recalls were recorded on 2 occasions, 2–4 weeks apart, by a registered dietician using Nutrition Data System software [26]. Actiwatch accelerometers (Mini Mitter Co, Inc, Bend, OR) were used to measure frequency, duration, and intensity of physical activity on 3 consecutive days [27].

#### Blood sampling and biochemical analyses

Blood samples were collected between 0700 and 0800 after a 12-hour overnight fast, centrifuged and aliquots of plasma and serum stored at −70°C for later analyses. Plasma methionine, total homocysteine (tHcy), tCys and total glutathione (tGSH) were measured by liquid chromatography tandem mass spectrometry [28]. Inter-assay coefficient of variation (CV) was <4% for tCys and tHcy, and <8% for methionine and tGSH.

Serum glucose, triglycerides, total cholesterol, and HDL-cholesterol were assayed by enzymatic-colorimetric techniques with the GM7 Analyzer (Analox Instruments, Lundeburg, MA) and Microquant Platereader (Biotek Instruments, Winooski, VT). NEFA (CV 2.7%) were determined using acyl-CoA synthetase and acyl-CoA oxidase supplied by Wako Chemicals.

Serum C-reactive protein (CRP), MCP-1, IL-6 and TNF-α were measured by ELISA as detailed previously [29]. Leptin (CV 8.3%), adiponectin (CV 3.6%) and insulin (CV 10%) were measured using a radioimmunoassay kit (Linco Research Inc, St Charles, MO).

The homeostasis model of insulin resistance (HOMA-IR) was calculated as (fasting insulin [µU/mL] * fasting glucose [mmol/L]/22.5), and used as a measure of insulin sensitivity. Children with HOMA-IR >3.16 were considered insulin resistant [30].

### Statistical Methods

Population characteristics are summarized as median (5^th^–95^th^ percentile). Group comparisons were conducted using Mann Whitney U test. P<0.05 was considered significant.

#### Interactions by gender

Using generalized additive linear models, we tested whether the associations of interest were modified by gender. We found significant interactions by gender in the associations of age with body composition and with plasma methionine and tGSH (but not tCys or tHcy). We therefore present these associations stratified by gender. There was no meaningful interaction between tCys and gender in predicting body fat%, HOMA-IR or inflammatory markers, so for these analyses, we present results pooled for girls and boys and adjusted for gender.

#### Correlation and regression analysis

Pearson correlation analysis and multivariate linear regression models were used. Using step-wise linear regression, we found no independent effect of Tanner stage, after controlling for age, in models investigating the association of tCys with body fat%, insulin resistance, and inflammatory markers, so Tanner stage was not included as a covariate. Analysis was repeated after taking into account clustering of data within families, with similar results. We report the analysis without adjusting for family clustering, since it gave more conservative estimates.

Logistic regression was used to calculate odds ratios for associations of tCys with obesity and insulin resistance.

#### Dose-response curves

We used generalized additive model (GAM) plots in package “mgcr” in R [31] to depict dose-response relations among age, body composition and SAA. At approximately mean exposure of the independent variable, the model generates a reference value of zero for the dependent variable. P-values and partial correlation coefficients were calculated from linear regression analyses.

Skewed variables were log-transformed prior to parametric analysis and dose-response modelling. All analyses except for dose-response curves were done using PASW Statistics for WINDOWS (18.0; SPSS Inc., Chicago, IL, USA).

## Results

### Anthropometric Parameters

Table-1 shows the population distribution of the variables of interest. The study included 489 boys, of whom 364 (74%) were overweight/obese, and 495 girls, 332 (67%) of whom were overweight/obese. Among normal-weight children, median CDC BMI percentile was also shifted to the right (62^nd^ percentile in boys; 64^th^ percentile in girls). As expected, adiposity parameters differed between normal-weight and overweight/obese children. Overweight/obese boys and girls were also taller than normal-weight children.

**Table 1:** Characteristics of the study population.^a^

			Boys				Girls				
		AllN = 489	Normal-weightN = 125	OW/ObeseN = 364	AllN = 495		Normal-weightN = 163			OW/obeseN = 332	
**Age, y**	11.3 (4.8–17.8)**^b^**		10.5 (4.3–17.6)	11.4 (5.3–18.0)		10.8 (4.4–17.8)		10.2 (4.3–17.8)	11.0 (4.6–17.8)		
**Weight, kg**	55 (21–114) **^c^**		36 (17–72)	65 (24–118) **^e^**		50 (18–94)		33 (16–59)	59 (22–99)**^e^**		
**Height, cm**	148 (110–175) **^c^**		141 (105–177)	150 (113–174) **^d^**		144 (105–166)		136 (102–161)	147 (107–166)**^e^**		
**BMI, kg/m^2^**	24.9 (15.8–39.5) **^b^**		17.8 (14.8–23.6)	27.7 (18.2–40.6) **^e^**		23.6 (15.2–37.7)		17.3 (14.2–23.7)	26.8 (18.1–39.7) **^e^**		
**CDC–BMI percentile**	97^th^ (36^th^–100^th^) **^c^**		62^nd^ (21^st^–83^rd^)	99^th^ (88^th^–100^th^) **^e^**		94^th^ (26^th^–100^th^)		64^th^ (10^th^–83^rd^)	98^th^ (87^th^–100^th^) **^e^**		
**Waist, cm**	78 (53–112) **^c^**		61 (50–80)	85 (58–114) **^e^**		71 (50–102)		58 (48–73)	79 (55–106) **^e^**		
**Waist-height ratio**	0.53 (0.41–0.70) **^c^**		0.44 (0.39–0.50)	0.56 (0.47–0.72) **^e^**		0.51 (0.40–0.67)		0.44 (0.39–0.49)	0.54 (0.47–0.69) **^e^**		
**Fat–free mass, kg**	36.4 (16.1–66.9) **^b^**		26.9 (13.5–57.4)	39.5 (18.3–67.8) **^e^**		31.8 (13.0–53.7)		23.6 (11.9–39.7)	35.6 (15.0–55.3) **^e^**		
**Fat mass, kg**	16.9 (4.2–42.5)		6.6 (3.3–15.7)	21.6 (6.0–44.7) **^e^**		17.2 (4.3–41.6)		8.6 (3.3–20.7)	23.0 (6.9–44.2) **^e^**		
**Trunk fat, kg**	6.94 (1.25–19.81)		2.17(1.03–5.81)	9.11 (1.98–20.64) **^e^**		7.53 (1.48–20.60)		3.01 (0.98–9.14)	10.33(2.56–21.71) **^e^**		
**Trunk–total fat ratio**	0.41 (0.29–0.50) **^c^**		0.33 (0.27–0.43)	0.43 (0.32–0.51) **^e^**		0.43 (0.32–0.52)		0.36 (0.29–0.46)	0.45 (0.36–0.53) **^e^**		
**Body fat%, %**	32.0 (15.0–46.0) **^c^**		20.0 (13.0–28.0)	36.0 (22.0–47.0) **^e^**		36.0 (21.0–47.0)		27.0 (19.0–36.0)	40.0 (29.0–48.0) **^e^**		
***Serum/Plasma Parameters***											
**Methionine, µmol/L**	23.8 (17.2–32.4) **^b^**		24.0 (16.3–33.8)	23.7 (17.5–32.4)		23.2 (17.3–30.1)		22.4 (17.2–28.5)	23.8(17.5–30.7) **^d^**		
**tHcy, µmol/L**	4.5 (3.0–7.4) **^c^**		4.5 (2.8–7.3)	4.6 (3.0–7.4)		4.1 (2.9–6.2)		4.1 (2.9–6.6)	4.1 (2.9–6.1)		
**tCys, µmol/L**	204 (159–253)		192 (151–232)	209 (163–255) **^e^**		201 (160–247)		192 (159–238)	205 (161–251) **^e^**		
**tGSH, µmol/L**	2.64 (1.73–4.72) **^c^**		3.04 (1.82–5.03)	2.54 (1.68–4.56) **^e^**		2.44 (1.69–4.00)		2.49 (1.72–4.40)	2.41 (1.66–3.66) **^d^**		
**Triglycerides, mg/dL**	91 (45–224)		70 (42–144)	103 (47–237) **^e^**		92 (45–202)		72 (42–156)	103 (51–220) **^e^**		
**NEFA, mmol/L**	0.49 (0.23–0.88)		0.48 (0.21–1.05)	0.50 (0.26–0.78)		0.51 (0.22–0.92)		0.50 (0.20–0.95)	0.51 (0.23–		
**Total protein, g/L**	73 (65–81)		72 (65–82)	73 (65–81)		73 (65–81)		73 (65–82)	72 (65–81)		
**Albumin, g/L**	44 (39–48)		44 (39–49)	43 (39–47)		43 (38–48)		44 (39–48)	43 (38–47)		
**Glucose, mg/dL**	93 (80–106) **^c^**		91 (78–103)	93 (82–107) **^e^**		90 (79–107)		88 (79–100)	92 (80–108) **^e^**		
**Insulin, µU/mL**	16.3 (5.0–60.2)		9.1 (3.8–23.1)	19.5 (6.4–62.4) **^e^**		17.2 (4.6–65.4)		10.0 (3.8–25.8)	22.7 (5.5–70.6) **^e^**		
**C**–**Peptide, ng/mL**	2.10 (0.60–6.40)		1.40 (0.50–3.20)	2.60 (0.62–6.60) **^e^**		2.33 (0.60–6.70)		1.50 (0.50–3.80)	2.90 (0.70–7.20) **^e^**		
**HOMA**–**IR index**	3.76 (1.07–14.38)		2.06 (0.77–5.45)	4.61 (1.45–15.01) **^e^**		3.84 (0.99–15.11)		2.24(0.79–5.69)	5.10 (1.15–17.67) **^e^**		
**Adiponectin, µg/L**	11.8 (4.6–23.5) **^b^**		14.8 (6.6–26.4)	10.3(4.4–22.0) **^e^**		12.9 (5.3–25.4)		15.9(8.2–30.4)	11.7(4.8–23.1) **^e^**		
**Leptin, ng/mL**	11.8 (2.0–40.4) **^c^**		3.3 (1.6–9.3)	16.0 (3.4–43.4) **^e^**		17.9 (3.0–49.4)		6.9 (2.4–23.4)	23.8 (4.8–57.3) **^e^**		
**CRP, ng/mL**	930 (45–4184)		131 (23–1430)	1246 (116–5234) **^e^**		821 (55–4394)		230 (35–1768)	1171 (106–5336) **^e^**		
**IL**–**6, pg/mL**	1.37 (0.53–5.88) **^b^**		0.98 (0.45–5.88)	1.50 (0.63–5.66) **^e^**		1.51 (0.62–6.33)		1.28 (0.55–6.49)	1.61 (0.67-5.83) **^d^**		
**MCP-1, pg/mL**	307 (147–521)		304 (156–491)	308 (146–541)		300 (151–529)		289 (148–488)	303 (155–543)		
**TNF**–**α, pg/mL**	8.4 (4.7–12.8)		8.5 (5.2–13.0)	8.4 (4.6–12.8)		8.1 (4.6–12.8)		8.4 (4.0–13.1)	8.1 (4.7–12.5)		

Abbreviations: OW, overweight; tHcy, total homocysteine; tCys, total cysteine; tGSH, total glutathione; HOMA-IR, homeostasis model assessment of insulin resistance; NEFA, non-esterified fatty acids; CRP, C-reactive protein; IL-6, interleukin-6; MCP-1, monocyte chemoattractant protein-1; TNF-α, tumor necrosis factor-alpha.

a Data presented as median (5–95%).

b, c Boys significantly different from girls at *P*<0.05 and *P*≤0.001, respectively.

d, e Normal-weight individuals significantly different from overweight/obese subjects within the same gender at *P*<0.05 and *P*≤0.001, respectively.

**Figure 1:**
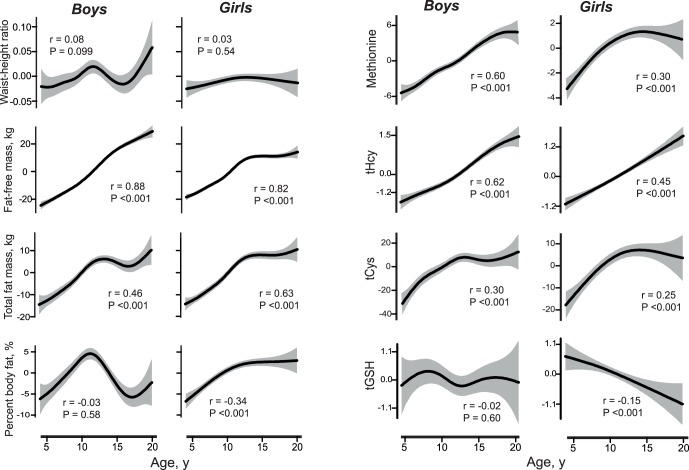
Age, body composition and sulfur amino acids. Dose-response curves (solid lines) with 95% confidence intervals (shaded area) for the association of age with body composition parameters (A) and with plasma concentrations (µmol/L) of methionine, tHcy (total homocysteine), tCys (total cysteine) and tGSH (total glutathione). Figures were constructed using generalized additive regression models in R (see Methods section for details). At approximately mean exposure of the independent variable, the model generates a reference value of zero for the dependent variable. *P*-values and correlation coefficients were obtained from corresponding Pearson correlation analysis.

Changes in body composition with age are shown in Figure-1. Waist-height ratio showed little variation with age. By definition, BMI Z-score and CDC-BMI percentile (not shown) were also independent of age. In contrast, mean BMI increased from age 4 to 19 years by 17 kg/m^2^ and 13 kg/m^2^ in boys and girls, respectively. The difference between boys and girls appeared to be explained by a relative tapering in growth of FFM and FM at around 11-12 years of age in girls, while growth of FFM, but not FM, continued linearly in boys. This resulted in a striking difference in change of body fat % between the genders, which started to decrease at that age in boys but not in girls (*P*_interaction_ <0.001).

### Plasma Sulfur Amino Acids

Methionine, tHcy and tGSH were marginally higher in boys than girls, while there was no difference in tCys. Overweight/obese girls and boys had higher tCys and lower tGSH compared to normal- weight children (Table-1).

Increasing age was associated with increases in plasma methionine, tHcy and tCys (Figure-1). Methionine reached a plateau after about age 12 years in girls, but continued to increase linearly in boys (*P*_interaction_ <0.001). Plasma tGSH decreased with age in girls but not in boys (*P*_interaction_ = 0.034).

### Associations Among Sulfur Amino Acids and Body Composition

Methionine, tHcy, tCys and tGSH were positively correlated with each other after adjustment for age and gender (Table-2), with the exception of lack of correlation between plasma tCys and tGSH. The strongest association was between tHcy and tCys (partial r = 0.40). Methionine, tHcy and tCys were positively associated with FFM, and tHcy showed a modest inverse association with body fat% (Table-2). tGSH correlated negatively with FFM, FM, body fat% and BMI Z-score.

**Table 2:** Correlations among the sulfur amino acids and body composition.^a^

	Methionine	tHcy	tCys	tGSH
**tHcy** b	**0.17**			
**tCys**	**0.13**	**0.40**		
**tGSH** b	**0.07** c	**0.18**	0.04	
**BMI Z-score**	0.05	−0.02	**0.29**	**−0.21**
**Body fat%**	−0.06	**−0.08** c	**0.30**	**−0.23**
**Fat mass**	0.00	−0.05	**0.31**	**−0.25**
**Fat-free-mass**	**0.18**	**0.08** c	**0.24**	**−0.17**

Abbreviations: tHcy, total homocysteine; tCys, total cysteine; tGSH, total glutathione.

a Pearson correlation coefficients adjusted for age and gender. Bold entries indicate significant correlations (*P*<0.001 unless otherwise stated).

b Using log-transformed data.

c *P*<0.05.

### Association of tCys with Body Fat

Plasma tCys was positively associated with FM and FFM (Table-2). After adjustment for FM, the association with FFM disappeared (partial r = 0.02, *P = *0.56), while after adjustment for FFM, the association with FM remained (partial r = 0.21, *P*<0.001).

Using linear regression adjusted for age and gender with body fat% as dependent variable, tCys explained 9% of the variability in body fat% (partial r = 0.30, *P*<0.001). The association remained robust after adjusting for plasma methionine, tHcy and tGSH (partial r for tCys = 0.37, *P*<0.001), or for plasma albumin and protein (not shown). Further adjustment for height, FFM, physical activity and dietary protein and fat intakes did not affect the association (partial r = 0.31, *P*<0.001). To test whether tCys is associated with central fat, we repeated the analysis using trunk fat/total fat ratio as the dependent variable. The partial r was 0.24 (*P*<0.001) with adjustment for age and gender, and 0.21 (*P*<0.001) in the fully adjusted model.

Due to substantial gender-specific changes in body fat% with age (Figure-1), we repeated the analysis using BMI Z-score and waist-height ratio as dependent variables. Both were fairly independent of age and gender. In the fully adjusted model (excluding height as a covariate), tCys explained nearly 7% of variability of waist-height ratio (partial r = 0.26, *P*<0.001), and 6% of BMI Z-score (partial r = 0.24, *P*<0.001). In separate models that did not include macronutrient intakes, we adjusted for dietary cystine and methionine intakes but these had negligible effects on the results.

**Figure 2:**
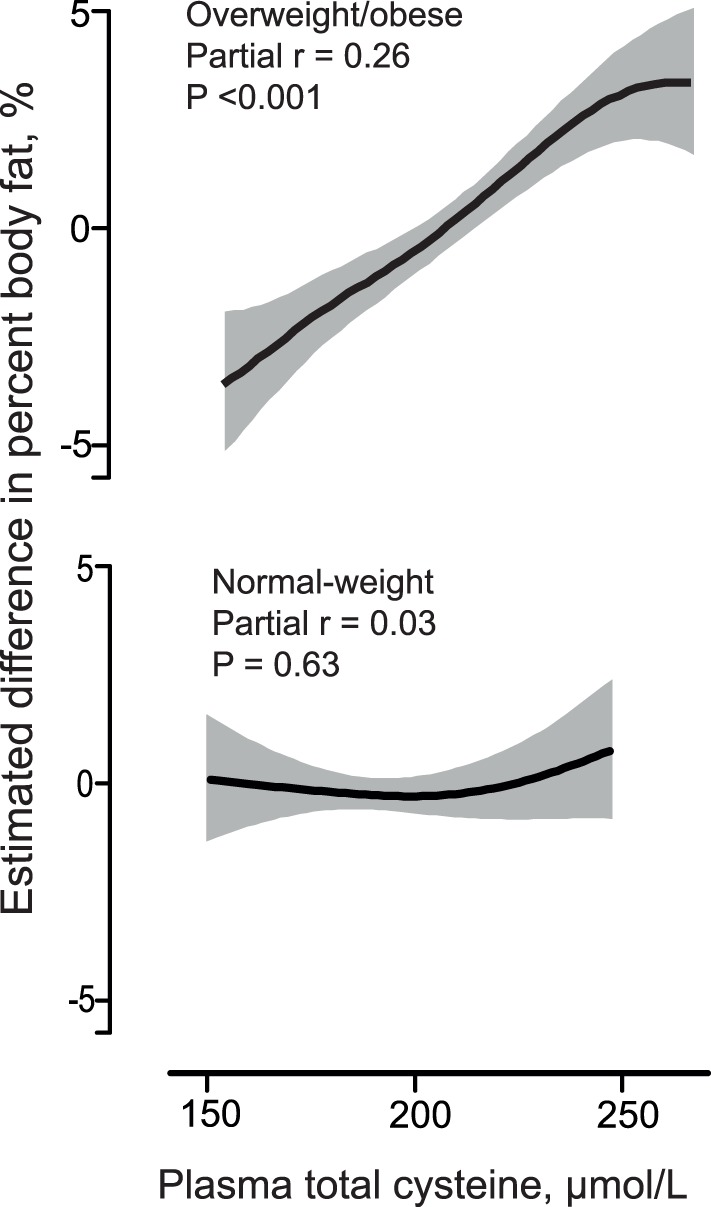
Association of tCys with body fat%. Dose-response curves (solid lines) with 95% confidence intervals (shaded area) for the association of plasma tCys with body fat% in normal weight (CDC-BMI percentile <85^th^; N = 288) and overweight/obese (CDC BMI percentile ≥85^th^; N = 696) children and adolescents after adjustment for age and gender. P-values and partial correlation coefficients were obtained from corresponding linear regression analysis. Data is shown only for the 2.5^th^–97.5^th^ percentiles of tCys for each group.

Since tCys was higher in overweight/obese children than in normal-weight children, we tested whether the association of tCys with body fat% varied between the 2 groups. tCys was associated with body fat% in overweight/obese children (partial r = 0.26, *P*<0.001; age and gender-adjusted), but not in normal-weight children (partial r = 0.03, *P* = 0.63; *P*_interaction_ = 0.002), despite overlapping tCys values (Figure-2). In the overweight/obese subgroup, after adjusting for other plasma SAA, serum lipids, as well as dietary protein and fat intakes and physical activity, tCys accounted for 9% of body fat% variability (partial r = 0.30, *P*<0.001). A similar discrepancy was observed in the relation between tCys and trunk fat/total fat ratio, where they were associated only in the overweight/obese group (partial r = 0.18, *P*<0.001; age- and gender-adjusted), but not the normal-weight group (partial r = 0.00, *P* = 1.0; *P*_interaction_ = 0.023).

**Figure 3:**
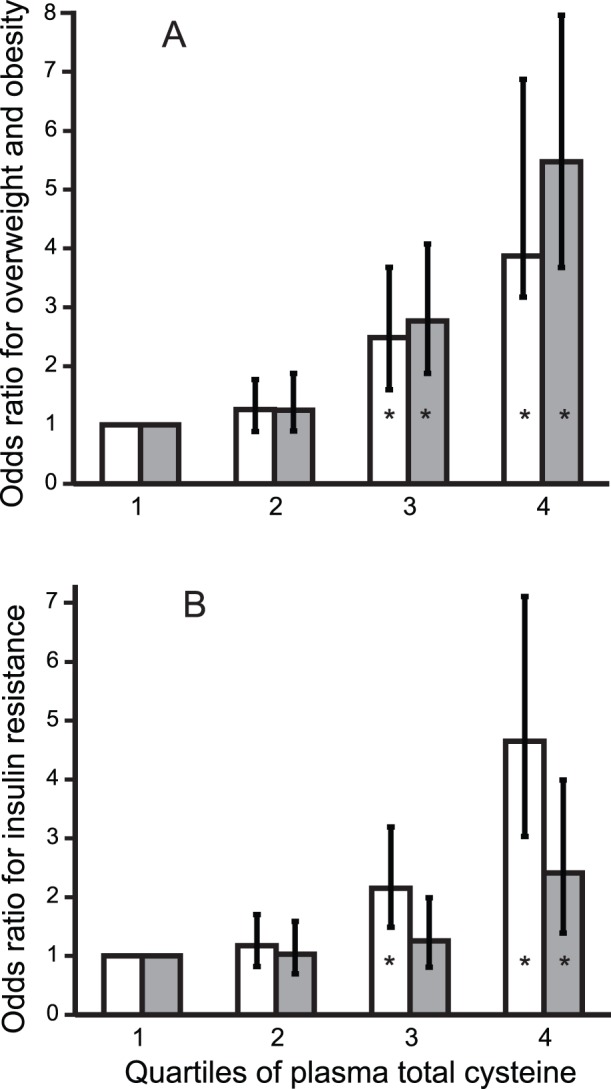
Odds ratios for overweight, obesity and insulin resistance according to plasma total cysteine. A: Age and gender-adjusted odds ratios and 95% confidence intervals for overweight (white bars) and obesity (grey bars) according to quartiles of plasma total cysteine (tCys). B: Age and gender-adjusted odds ratios for insulin resistance (defined as HOMA-IR index >3.16) by quartiles of plasma tCys, with (grey bars) and without (white bars) adjustment for body fat%. *P≤0.001.

We tested whether plasma tCys was associated with allocation to normal weight, overweight or obese categories. In separate multiple logistic regression models adjusted for age and gender, children in upper versus lowest tCys quartile were 4-times as likely to be overweight and 5-times as likely to be obese (*P*<0.001 for both; Figure-3-A). As observed in the linear models (above), the risk estimates were robust to adjustment for possible confounders (data not shown).

### Association of tCys with Inflammatory Markers and Insulin Resistance

As expected, HOMA-IR and pro-inflammatory markers, leptin, CRP and IL-6 were higher in overweight/obese children. Overall, boys had, compared to girls, lower levels of adiponectin, leptin, and IL-6; Table-1).

No associations were observed between tCys and IL-6, MCP-1 or TNF-α (Table-3, Model-1). tCys was positively associated with CRP independently of other SAA (Model-1), but this association was abolished by adjusting for BMI Z-score (Model-2) or body fat% (Model-3). tCys showed a strong positive association with serum leptin that was partly independent of other SAA and body composition. Replacing body fat% in Model-3 with FM gave essentially similar results (not shown).

**Table 3:** Association of plasma tCys with circulating inflammatory markers, adipokines and insulin-resistance-related parameters.^a^

	Model 1N = 950	Model 2N = 949	Model 3N = 929
**Leptin** b	0.36 (*P<0.001)*	0.20 *(P<0.001)*	0.10 *(P = 0.001)*
**CRP** b	0.21 (*P<0.001)*	0.05 (*P = 0.14)*	0.01 (*P = 0.82)*
**IL-6** b	0.02 *(P = 0.48)*	−0.04 *(P = 0.25)*	−0.06 (*P = 0.077)*
**MCP-1** b	0.03 *(P = 0.36)*	0.01 *(P = 0.83)*	−0.00 (*P = 0.94)*
**TNF-α**	−0.06 *(P = 0.061)*	−0.05 *(P = 0.092)*	−0.05 *(P = 0.10)*
**Glucose** b	0.18 *(P<0.001)*	0.13 *(P<0.001)*	0.13 *(P<0.001)*
**Insulin** b	0.27 *(P<0.001)*	0.11 *(P<0.001)*	0.06 *(P = 0.080)*
**HOMA-IR** b	0.29 *(P<0.001)*	0.13 *(P<0.001)*	0.08 *(P = 0.014)*
**C-Peptide** b	0.22 *(P<0.001)*	0.06 *(P = 0.06)*	0.02 *(P = 0.57)*
**Adiponectin**	−0.14 *(P<0.001)*	−0.06 *(P = 0.43)*	−0.03 *(P = 0.39)*
**NEFA** b	0.15 *(P<0.001)*	0.13 *(P<0.001)*	0.10 *(P = 0.002)*

Abbreviations: CRP, C-reactive protein; IL-6, interleukin-6; MCP-1, monocyte chemoattractant protein-1; TNF-α, tumor necrosis factor-alpha; HOMA-IR, homeostasis model assessment of insulin resistance; NEFA, non-esterified fatty acids.

a Pearson correlation coefficients, adjusted for the following covariates:

Model 1: age, gender, plasma methionine, total homocysteine (tHcy) and total glutathione (tGSH).

Model 2: as in Model 1+ BMI-Z-score.

Model 3: as in Model 1+ body fat%.

b Using log-transformed data.

In analysis stratified by weight group and adjusted for age and gender, tCys was associated with leptin (partial r = 0.25, *P*<0.001) and CRP (partial r = 0.12, *P* = 0.002) only in the overweight/obese subgroup, but not in normal-weight children. TNF-α and IL-6 were not associated with tCys in either subgroup (data not shown).

tCys was positively associated with fasting glucose, insulin, C-peptide, NEFA and HOMA-IR, and negatively associated with adiponectin (Table-3, Model-1), independent of other SAA. The associations were markedly weakened by adjustment for BMI Z-score (Model-2), body fat% (Model-3) or FM (not shown). In Model-3, tCys remained modestly associated with fasting glucose (partial r = 0.13, *P*<0.001) and HOMA-IR (partial r = 0.08, *P* = 0.019).

In analysis stratified by weight group, tCys correlated with HOMA-IR more strongly in overweight/obese children (partial r = 0.20, *P*<0.001) than in normal-weight children (partial r = 0.09, *P* = 0.15) in age- and gender-adjusted analysis (*P*_interaction = _0.027).

Using logistic regression adjusted for age and gender, we tested whether high tCys was associated with insulin resistance. Children in the upper tCys quartile were 4 times as likely to have a HOMA-IR >3.16 [30], compared to those in the first quartile (*P*<0.001; Figure-3-B). After controlling for body fat%, high tCys remained associated with a 2-fold risk of insulin resistance.

## Discussion

In view of the relation of plasma tCys with obesity in European adults [12], [13], we investigated whether tCys is associated with inflammatory obesity and insulin resistance in Hispanic children. tCys was independently associated with body fat% and markers of central adiposity, namely waist-height ratio and trunk fat to total fat ratio. High tCys was associated with a 2-fold risk of having insulin resistance, independent of body fat. tCys was not was not consistently associated with markers of systemic inflammation. However, it was positively associated with CRP, an inflammatory marker and independent predictor of cardiovascular disease [32].

### Associations Among Age, Plasma Sulfur Amino Acids and Body Composition

As observed previously, plasma methionine, tHcy and cysteine increased with age [33], [34]. We observed that tCys in both genders and methionine in girls reached a plateau around puberty. The continued increase in methionine in boys beyond puberty may relate to their enhanced accretion of muscle mass, which is suggested to be an important determinant of plasma methionine [35]. A marked gender-specific effect of age on body fat% was observed. Body fat% increased in boys and girls from age 4 years till about 11 years; then reached a plateau in girls. In boys, body fat% decreased, concomitant with continued increase in FFM but not FM. Molgaard et al previously noted this difference in fat% after puberty [36].

Plasma methionine, tHcy and tCys were positively correlated, as expected from their metabolic link and previously seen in adults [15]. tGSH was inversely associated with body fat% and BMI Z-score and was lower in overweight/obese girls and boys compared to normal-weight children. Our findings in children are consistent with reports that reduced glutathione is decreased in obese adults [37]. Increased oxidative stress resulting from the obese state is believed to underlie these associations [38].

### tCys in Relation to Adiposity, Insulin Resistance and Inflammatory Markers

Independent of concentrations of other sulfur compounds, plasma tCys was associated with all measures of adiposity, accounting for 9% and 6% of the variability in body fat% and BMI Z-score respectively. Children in the upper tCys quartile had a 5-fold higher risk of being obese, compared to those in the first quartile. The finding of this association in children of non-European origin suggests that tCys may be a determinant of FM across ages and ethnic groups.

Several additions and differences to previous reports of the tCys-obesity association [12], [13] should be noted, including the independence of the association from plasma albumin, which binds most of the cysteine in plasma. Also, tCys was associated with insulin resistance, where children in the upper tCys quartile were 4 times as likely to have a HOMA-IR index above 3.16. This was not totally mediated by obesity, since adjustment for body fat% left a substantial residual association (2-fold risk for upper tCys quartile). tCys was further correlated with serum NEFA, which play an important role in development of insulin resistance in obesity [39].

We screened several adipokines for possible relationships with plasma tCys. Leptin and adiponectin are the most abundant cytokines produced by adipocytes and link obesity with inflammation and insulin resistance [40]. Methionine restriction in rats lowers serum leptin and raises adiponectin, while cysteine supplementation reverses these effects [8]. In the present study, tCys showed strong positive and negative associations with leptin and adiponectin, respectively, consistent with the rat findings [8]. TNF-α, IL-6, MCP-1 and CRP are elevated in obese individuals, and predict diabetes and cardiovascular disease [40]. tCys was positively related to CRP, but not to TNF-α, IL-6, or MCP-1. Thus tCys appeared to be associated only with the inflammatory markers that strongly correlate with BMI and fat mass, including CRP and leptin. Indeed the association of tCys with CRP was totally mediated by body fat.

Interestingly, the positive relation of tCys with total body fat% and central obesity with insulin resistance was observed only in overweight/obese children but not in normal-weight children and adolescents. This may have been caused by low power in the normal-weight group, which was smaller (N = 288) than the overweight/obese (N = 696) group. However, the effect size estimates and the tCys-fat% dose-response curve suggest a true lack of association. In contrast, in our study of >5000 adults [12], tCys correlated with FM both in normal weight and overweight/obese individuals. This suggests that tCys may be associated with body fat via a factor that is present in adults and overweight/obese children, but not in normal-weight children, such as large adipocyte size, with its distinct genetic and metabolic profiles [41], [42]. For example, expression and activity of SCD1, a recently identified target of cysteine action [8], are higher in large than in small adipocytes [43]. Obese children display earlier and faster increases in size and number of adipocytes, reaching levels seen in lean adults by 11 years of age [44]. Another possible explanation is that adults have higher tCys concentrations, ranging approximately from 200–400 µM [12], while tCys values in the children in the present cohort were between 150 and 260 µM. If cysteine exerts an obesogenic action only at relatively high levels, it is possible that at low tCys concentrations not all individuals are susceptible to this action.

### Conclusions

In summary, plasma tCys was associated with body fat% and insulin resistance in Hispanic children and adolescents, extending the previously reported association of tCys with obesity in European adults [12], [13]. The association with insulin resistance was partly independent of body fat, but was not related to proinflammatory cytokines TNF-a, IL-6, or MCP-1. The cross-sectional design of this study precludes inferences about causality, but the findings are in line with the effect of dietary cysteine in inducing diabetogenic enzymes and promoting adiposity and glucose intolerance in some rodent models [8], [9]. Prospective studies are needed to determine whether elevated tCys could be a useful marker for predicting future weight gain, insulin resistance, diabetes or clinical outcomes. Together with recent evidence from knockout mice lacking functional enzymes involved in SAA metabolism, our findings in humans highlight a previously underappreciated link between the SAA pathway and obesity and cardiometabolic risk.

## Supporting Information

Figure S1 **Enzymes in the sulfur amino acid pathway that are related to body weight/composition in mice.** Knockouts of the enzymes shown in bold are associated with prominent body weight changes. BHMT [2] and GCL modifier subunit (GCLM) [3] knockouts have high metabolic rate and insulin sensitivity and resist obesity. CBS knockouts have markedly decreased body fat [1]. CGL and GGT knockouts have low body weight that is reversed by L-cysteine [45] or N-acetylcysteine [46] supplementation. Common to all 5 knockouts is decreased plasma cyste(i)ne [3], [46] or tCys [1], [6], [45]. Hepatic expression of stearoyl coenzyme A desaturase, a lipid enzyme that is considered a key checkpoint in development of obesity [7] was investigated in CBS and GCLM knockouts and found to be decreased [1], [4]. BHMT, betaine homocysteine methyltransferase; CBS, cystathionine beta synthase; CGL, cystathionine gamma lyase; GCL, glutamate-cysteine ligase; GGT, gamma-glutamyltransferase; MS, methionine synthase; CDO, cysteine dioxygenase. Dotted lines show pathways with omitted intermediates for purposes of clarity.
(EPS)
